# SLAH1, a homologue of the slow type anion channel SLAC1, modulates shoot Cl^−^ accumulation and salt tolerance in *Arabidopsis thaliana*

**DOI:** 10.1093/jxb/erw237

**Published:** 2016-06-23

**Authors:** Jiaen Qiu, Sam W Henderson, Mark Tester, Stuart J Roy, Mathew Gilliham

**Affiliations:** ^1^School of Agriculture, Food, and Wine, University of Adelaide, PMB1, Glen Osmond, SA 5064, Australia; ^2^Australian Centre for Plant Functional Genomics, PMB1, Glen Osmond, SA 5064, Australia; ^3^ARC Centre of Excellence in Plant Energy Biology, PMB1, Glen Osmond, SA 5064, Australia; ^4^Centre for Desert Agriculture, King Abdullah University of Science and Technology, Thuwal 23955–6900, Kingdom of Saudi Arabia

**Keywords:** ABA, Arabidopsis, AtSLAH1, AtSLAH3, chloride, Cl^−^ xylem loading, long-distance transport, nutrition, salinity, slow-type anion channel-associated homologue 1, slow-type anion channel-associated homologue 3.

## Abstract

Salinity tolerance is correlated with shoot chloride (Cl^–^) exclusion in multiple crops, but the molecular mechanisms of long-distance Cl^–^ transport are poorly defined. Here, we characterize the *in planta* role of AtSLAH1 (a homologue of the slow type anion channel-associated 1 (SLAC1)). This protein, localized to the plasma membrane of root stelar cells, has its expression reduced by salt or ABA, which are key predictions for a protein involved with loading Cl^–^ into the root xylem. Artificial microRNA knockdown mutants of *AtSLAH1* had significantly reduced shoot Cl^−^ accumulation when grown under low Cl^–^, whereas shoot Cl^–^ increased and the shoot nitrate/chloride ratio decreased following *AtSLAH1* constitutive or stelar-specific overexpression when grown in high Cl^–^. In both sets of overexpression lines a significant reduction in shoot biomass over the null segregants was observed under high Cl^–^ supply, but not low Cl^–^ supply. Further *in planta* data showed *AtSLAH3* overexpression increased the shoot nitrate/chloride ratio, consistent with AtSLAH3 favouring nitrate transport. Heterologous expression of *AtSLAH1* in *Xenopus laevis* oocytes led to no detectible transport, suggesting the need for post-translational modifications for AtSLAH1 to be active. Our *in planta* data are consistent with AtSLAH1 having a role in controlling root-to-shoot Cl^–^ transport.

## Highlight

Manipulation of AtSLAH1 expression modifies shoot Cl^–^ accumulation and salt tolerance in Arabidopsis, consistent with its proposed role in regulating Cl^–^ transport from root to shoot.

## Introduction

Chloride (Cl^–^) is classified as a micronutrient, but it is often present in plant tissues at concentrations typical of a macronutrient (i.e. 2–20 rather than 0.1–200 μg g^–1^ DW) (Marschner, 1995; Xu *et al.*, 2000; Broadley *et al.*, 2012; Franco-Navarro *et al.*, 2015). Cl^–^ has vital roles in regulating numerous physiological processes including turgor, enzyme activity, photosynthesis and membrane potential (Rognes, 1980; White and Broadley, 2001; Teakle and Tyerman, 2010). Although the pathways for Cl^–^ entry and movement within the plant have been characterized biochemically, their molecular determinants are poorly defined (Teakle and Tyerman, 2010; Henderson *et al.*, 2014).

High concentrations of sodium chloride (NaCl) in soils reduces crop yield (Rengasamy, 2010, Roy *et al.*, 2014), which can impose significant economic costs to farmers (Munns and Gilliham, 2015). Na^+^ transport and its impact on plant growth have been relatively well documented at both a physiological and a molecular level in a variety of plant species (Blumwald *et al.*, 2000; Zhu, 2003; Horie and Schroeder, 2004; Davenport *et al.*, 2005; Garthwaite *et al.*, 2005; Chinnusamy *et al.*, 2006; Apse and Blumwald, 2007; Horie *et al.*, 2008; Müller *et al.*, 2014; Roy *et al.*, 2014; Maathuis *et al.*, 2014; Flowers *et al.*, 2015). However, in other economically important crop plants like soybean, grapevine, citrus and lotus, leaf Cl^–^ accumulation (not Na^+^) is correlated with decreased plant growth and photosynthesis when plants are under salt stress (Storey and Walker, 1999; Walker *et al.*, 2002; Tregeagle *et al.*, 2006; Teakle *et al.*, 2007; Teakle and Tyerman, 2010; Gong *et al.*, 2011). So, although Cl^–^ is a micronutrient it can also accumulate to concentrations that inhibit plant growth when plants encounter salinity. The cause of both Na^+^- and Cl^–^-induced reductions in photosynthesis and growth, and the cause of salt-induced cell death are yet to be definitively determined and are a priority area for research (Munns and Gilliham, 2015). Some studies have investigated the relative impact of Na^+^ and Cl^–^ on barley and wheat (e.g. Tavakkoli *et al.*, 2011; Genc *et al.*, 2015). Growth and photosynthesis of several cultivars of barley appeared to be more sensitive to the addition of Cl^–^ than of Na^+^ (Tavakkoli *et al.*, 2011). These findings highlight the importance of investigating the regulation of both Na^+^ and Cl^–^ transport for improving plant salt tolerance, even in species that are classically considered to be more Na^+^-sensitive than Cl^–^-sensitive under saline conditions.

Identification of genes that underpin root-to-shoot Cl^–^ transport, and the related signalling pathways, should provide information that could be used to reduce Cl^–^ sensitivity in commercial crops. A key pathway in controlling Cl^–^ accumulation in the shoot is its loading from xylem parenchyma cells into the transpiration stream (Teakle and Tyerman, 2010). Recently, a nitrate (NO_3_^–^) transporter 1/peptide transporter family member (*NPF2.4*) was identified as the first protein to be directly involved in loading Cl^–^ into the root xylem (Li *et al.*, 2016). However, Cl^–^ accumulation in the shoot is predicted to be a multigenic trait in a number of plant groups and species including soybean, maize, grapevine, citrus and legumes (Abel, 1969; Storey and Walker, 1999; Moya *et al.*, 2003; Sibole *et al.*, 2003; Gilliham and Tester, 2005; Gong *et al.*, 2011; Henderson *et al.*, 2014; Fort *et al.*, 2015). Knockouts of *Atnpf2.4* had a 20% reduction in shoot Cl^–^ (Li *et al.*, 2016), providing further evidence of the multigenic nature of shoot Cl^–^ accumulation. Therefore, other anion transport proteins are likely to be involved in root-to-shoot Cl^–^ transport (e.g. Henderson *et al.*, 2014), but these remain to be identified and functionally characterized at a molecular level.

Three anion conductances have been identified using electrophysiology in barley root xylem parenchyma protoplasts, namely an inwardly rectifying anion channel (X-IRAC), a quickly activating anion conductance (X-QUAC) and a slowly activating anion conductance (X-SLAC) (Köhler and Raschke, 2000). Similar results were found in maize root stelar cells (Gilliham and Tester, 2005) and Arabidopsis root pericycle cells (Kiegle *et al.*, 2000). X-QUAC is the most prevalent conductance observed in xylem parenchyma cells and is likely to load the majority of Cl^–^ (and NO_3_^–^) ions into the xylem under non-saline conditions (Köhler *et al.*, 2002; Gilliham and Tester, 2005) as the estimated flux through this conductance could easily account for the Cl^–^ release from the xylem vessels measured using a ^36^Cl^–^ tracer (Pitman, 1982; Köhler and Raschke, 2000).

The hormone abscisic acid (ABA) regulates solute transport from root to shoot (Cram and Pitman, 1972). Excised barley roots treated with ABA for 2h accumulated significantly more Cl^–^ than untreated roots (Cram and Pitman, 1972). Cl^–^ efflux to the xylem was also reduced following the ABA treatment, but Cl^–^ influx into the root was unaffected (Cram and Pitman, 1972). These results indicate that ABA down-regulates xylem loading of Cl^–^ in roots but not root Cl^–^ influx. Furthermore, the anion conductances in maize, barley and Arabidopsis stele, as well as the potassium conductance through the stelar K^+^ outwardly rectifying channel (SKOR), are also down-regulated by ABA (Cram and Pitman, 1972; Gilliham and Tester, 2005). In Arabidopsis, *AtSKOR* was transcriptionally down-regulated by ABA (Gaymard *et al.*, 1998). Therefore, it may be possible to identify candidate genes for Cl^–^ loading into the root xylem by characterizing those genes encoding putative anion transporters that are expressed in the stele and are down-regulated (either transcriptionally or post-translationally) by ABA.

Early electrophysiological studies on stomatal guard cells revealed the slowly activated anion conductance (SLAC) (Linder and Raschke, 1992). More recently, the gene encoding the protein responsible for this conductance, *SLAC1*, was identified (Negi *et al.*, 2008; Vahisalu *et al.*, 2008). SLAC1 is a plasma membrane (PM) localized protein, highly permeable to malate and chloride (Negi *et al.*, 2008; Chen *et al.*, 2010), and *slac1* mutants have increased Cl^–^ in guard cells (Negi *et al.*, 2008; Vahisalu *et al.*, 2008). Four homologues of SLAC1, the slow-type anion channel-associated homologues 1 to 4 (SLAH1 to 4), have been identified that also localize to the PM and are predicted to be involved in anion transport (Negi *et al.*, 2008; Vahisalu *et al.*, 2008). Expression of *SLAH1* or *SLAH3* in *slac1* knockout mutants could complement both the defective closure response of *slac1* stomata to high CO_2_ and its ionic profile when constitutively overexpressed (Negi *et al.*, 2008). *SLAH3* is expressed in guard cells and roots, and is preferentially selective for NO_3_^–^ over Cl^–^; it has a role in NO_3_^–^ alleviation of ammonium toxicity (Geiger *et al.*, 2009, 2011; Demir *et al.*, 2013; Zheng *et al.*, 2015). *SLAH2* is also expressed in roots (Maierhofer *et al.*, 2014), and the protein is predominantly permeable to NO_3_^–^ (with a NO_3_^–^/Cl^–^ permeability ratio of 82) (Maierhofer *et al.*, 2014). Both SLAH2 and SLAH3 have been predicted to have roles in loading NO_3_^–^ into the root xylem (Maierhofer *et al.*, 2014; Zheng *et al.*, 2015). *SLAH1* is also expressed in the root; however, the role of AtSLAH1 is currently unknown. As *SLAH1* is expressed in the root stele and can complement the stomatal phenotype of the *Atslac1* mutant when ectopically expressed (Negi *et al.*, 2008; Vahisalu *et al.*, 2008; Zheng *et al.*, 2015), we examined whether AtSLAH1 has a role in loading Cl^–^ into the root xylem.

## Materials and methods

### Plant materials and growth conditions

All chemicals were obtained from Sigma-Aldrich unless stated. *Arabidopsis thaliana* ecotype (Col-0) seeds were purchased from the European Arabidopsis Stock Centre (Nottingham, UK). Plants were grown within temperature controlled growth rooms. Arabidopsis plants grown in soil were kept in long day conditions (16h day/8h night), while those in hydroponics were kept in short day conditions (10h day/14h night). In both long day and short day conditions, the temperature was maintained at 21–23 °C, the humidity was maintained between 60–75%, and the irradiance during the light period was 150 μmol m^–2^ s^–1^. Plants were grown in hydroponics following protocols described in Conn *et al.* (2013) and in soil following methods described in Møller *et al.* (2009).

### Generation of AtSLAH1 artificial microRNA lines

The *AtSLAH1* T-DNA knockout mutant (FLAG_329G06) was ordered through the Versailles Arabidopsis Stock Centre; however, the expression of *AtSLAH1* (At1G62280) was detectable in all homozygous mutant lines (Supplementary Fig. S1 at *JXB* online). To elucidate the function of AtSLAH1 *in planta*, artificial microRNAs (amiRNAs) were designed to knockdown *AtSLAH1* expression. To produce *AtSLAH1* knockdown mutants, specific amiRNAs were designed against the *AtSLAH1* mRNA sequence using Micro RNA Designer (http://wmd3.weigelworld.org/cgi-bin/webapp.cgi) following the protocol of Schwab *et al.* (2006). Two 21bp target sequences (TAAAACGCTATTTGGTTCCGT and TTATGTCTAGTGTCGAGACTG) were identified from the *AtSLAH1* coding sequence and two independent amiRNA constructs were generated with a set of primers (Supplementary Table S1) to incorporate the 21bp amiRNA sequence into the MIR319a vector (Schwab *et al.*, 2006). Both full-length *SLAH1*-amiRNA products were cloned using high-fidelity Phusion® polymerase (New England Biolabs, USA) into a Gateway® enabled pCR8 entry vector (Invitrogen, CA, USA) and transferred into the pMDC32 expression vector (Curtis and Grossniklaus, 2003) through an LR reaction (Invitrogen). The constructs were transformed into Arabidopsis using Agrobacterium-mediated floral dip transformation (Clough and Bent, 1998). Hygromycin B (20 μg ml^–1^) was used to select the transformants following the protocol described in Harrison *et al.* (2006).

### Generation of cell type-specific overexpression lines

An Arabidopsis enhancer trap line (E2568; Møller *et al.*, 2009) was used to generate plants with *AtSLAH1* root stelar-cell-specific overexpression. *AtSLAH1* full length cDNA was cloned using high-fidelity Phusion® polymerase (New England Biolabs, USA) from Arabidopsis root cDNA (following RNA extraction and cDNA synthesis following Henderson *et al.* (2015)) into a Gateway-enabled pCR8 entry vector (Life Technologies, CA, USA). *AtSLAH1* was then transferred into a pTOOL5 destination vector (pMDC132+UAS+NOS) (Plett, 2008) containing the GAL4-inducible promoter UAS, which drives target gene expression specifically in root stelar cells in line E2568. The construct was transformed into Arabidopsis line E2586 using Agrobacterium-mediated transformation (Clough and Bent, 1998). The seeds from transformed plants were harvested and germinated in soil. When the seedling had two to four true leaves, 20mg l^–1^ BASTA (Bayer, Germany) was sprayed on the seedlings to select for plants with the transgenic insertion.

### Generation of constitutive overexpression lines

Full-length *AtSLAH1* and *AtSLAH3* coding sequences were amplified (the primers used are listed in Supplementary Table S1) from Arabidopsis root cDNA, using Phusion® polymerase, cloned into the Gateway-enabled pCR8 entry vector (Life Technologies) and transferred into the pMDC32 expression vector (Curtis and Grossniklaus, 2003) through an LR reaction (Life Technologies). The construct was transformed into Arabidopsis (Col-0) using Agrobacterium-mediated transformation (Clough and Bent, 1998). Hygromycin B (20 μg ml^–1^) was used to select lines containing the transgene insertion following the protocol described in Harrison *et al.* (2006).

### Salinity and ABA treatment

Both salinity and ABA treatment were performed in hydroponics (Conn *et al.*, 2013). For the 7-day salinity treatment, NaCl was added to basal nutrient solution (BNS) (Conn *et al.*, 2013) to make a final concentration of 50, 75, and 100mM. Additional CaCl_2_ was added to each solution to achieve a constant Ca^2+^ activity of 1.3mM following the addition of high concentrations of monovalent cations, which act to reduce the activity of other cations and induce calcium deficiency (as detailed in Conn *et al.* (2013)). For ABA treatment, a stock solution of 100mM (±)-*cis*-*trans*-abscisic acid was made in absolute ethanol. When applying 20 µM ABA, this resulted in a final ethanol concentration of 0.01% (v/v) when added into the growth solution.

### Expression analysis

Gene expression analysis by qRT-PCR was performed following the method described in Burton *et al.* (2008). The primers for examining *AtSLAH1* expression were 5′TCTTCATGTCCCTGGTCTG3′ (forward) and 5′ATTGCTGTTTGCTGCTGTC3′ (reverse) and for *AtSLAH3* were 5′ATCTCTCGGTCGTTGGGAACTTTG3′ (forward) and 5′CTCGTTGGTCGGTAGCCTTTGG3′ (reverse). The selected Arabidopsis housekeeping genes (*AtGAPDH* (At3G26650), *AtActin2* (At3G18780), *AtTubulin* (At1G50010) and *AtCyclophilin* (At2G36130)) and data normalization followed the methods described in Jha *et al.* (2010). For relative gene expression, *AtActin2* was used as a control gene, and the relative expression level of target genes was detected using the same primer pairs as listed above using a QuantStudio 12K Flex Real-Time PCR system (Life Technologies).

### Phenotyping transgenic plants

For determining the shoot NO_3_^−^ concentration, a method that uses salicylic acid to form a chromophore with NO_3_^−^ under alkaline conditions (pH>12) was used (Cataldo *et al.*, 1975). In brief, 3–5mg of Arabidopsis dried tissue was extracted in 0.5ml deionized water, with 0.05ml of the extraction incubated with 0.2ml of 5% (w/v) salicylic acid–H_2_SO_4_ for 20min at room temperature; 0.05ml of this mixture was transferred into a fresh tube and 0.95ml of 2M NaOH was added. A 0.2ml aliquot was transferred to a well of a transparent 96-well plate and the absorbance at OD_410nm_ determined. A serial dilution of known concentrations of potassium nitrate (KNO_3_) was used for the standard, which ranged from 0 to 50mM.

For determining the shoot Cl^–^ concentration, 20–30mg of freeze-dried Arabidopsis tissue was extracted in 500 μl 1% nitric acid at 80 °C overnight. A chloride analyser (Model 926S, Sherwood Scientific, Cambridge, UK) was used to examine the Cl^–^ concentration by following the manufacturer’s instructions.

### Expression and electrophysiological characterization of AtSLAH1 in *X. laevis* oocytes

The *AtSLAH1*, *AtSnRK2.2*, and *AtSnRk2.3* coding sequences (primers are listed in Supplementary Table S1) were cloned using high-fidelity Phusion® polymerase (New England Biolabs, USA) from Arabidopsis root cDNA into a Gateway-enabled pCR8 entry vector (Life Technologies) before being transferred into the pGEM-HE (DEST) vector, and cRNA was synthesized using the mMESSAGE mMACHINE® kit (Ambion, Australia) as previously described (Preuss *et al*, 2011). Healthy stage IV–VI defoliculated oocytes were obtained through surgery and enzymatic digestion of ovaries from toads kept in an *Xenopus* colony at the Waite Campus (University of Adelaide). The cRNA (46 nl/23ng per oocyte) was injected using a micro injector (Drummond Nanoject II injector, USA) with a glass microcapillary pipette following the manufacturer’s procedures. The same volume of nuclease-free water was injected into control oocytes. Injected oocytes were incubated at 18 °C for 2 days in an ND96 solution (96mM NaCl, 2mM KCl, 1mM MgCl_2_, 5mM 4-(2-hydroxyethyl)-1-piperazineethanesulfonic acid (HEPES), 1.8mM CaCl_2_, pH 7.4 with 1M Tris) combined with horse serum (50 ml l^–1^), tetracycline (50 μg ml^–1^) and penicillin (50 μg ml^–1^). After 2 days, *AtSLAH1* cRNA-injected oocytes were voltage clamped from +40 to –120 mV in 20 mV decrements for 3s perfusing in the following bath solutions (basal: 2mM calcium gluconate, 5mM HEPES and 0.1mM LaCl_3_) plus 1 or 20mM CsNO_3_/CsCl at pH 7.5. Two-electrode voltage clamping (TEVC) was performed on oocytes as previously described in Roy *et al.* (2008) using an OC-725C amplifier (Warner Instruments Corp., USA), signals were digitized with a Digidata 1440A (Molecular Devices, USA), and then the data were recorded and analysed using pCLAMP 10.2 (Molecular Devices, USA).

## Results

### *AtSLAH1* expression is down-regulated by both salt and ABA

**Fig. 1.**
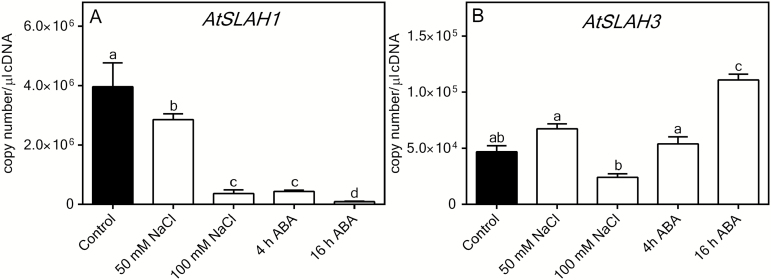
Expression level of *AtSLAH1* (A) and *AtSLAH3* (B) treated with control (2mM NaCl), 50mM and 100mM NaCl for 7 days, or 20 μM ± *cis*-*trans*-ABA for 4 or 16h. Arabidopsis (Col-0) were grown in hydroponics for 5 weeks and exposed to NaCl treatment for 7 days. The ABA was applied 4 or 16h before harvest. Transcripts were detected in the whole root cDNA. Results are presented as means+SEM, *n*=5. The expression levels were normalized to four control genes (*AtGAPDH*, *AtActin2*, *AtTubulin* and *AtCyclophilin*). Statistical significance was determined by one-way analysis of variance (ANOVA) and Tukey’s test (*P*≤0.05); a, b and c represent data groups that are statistically different from each other.

A qRT-PCR was performed on Arabidopsis root cDNA to determine whether *AtSLAH1* transcript abundance altered following salt or ABA treatment. The expression level of *AtSLAH1* was significantly reduced by 91% after 7 days of 100mM NaCl treatment, and by 97% after 16h of 20 µM ABA treatment when compared with the control (Fig. 1A). In contrast, the close homologue *AtSLAH3*, which shares the same cell location in xylem parenchyma and pericycle cells of the root stele and is also PM localized (Negi *et al.*, 2008), was not down-regulated by ABA or salt treatment (Fig. 1B). All these data are consistent with previous observations (Kilian *et al.*, 2007; Brady *et al.*, 2007; Gifford *et al.*, 2008; Supplementary Fig. S2).

### *AtSLAH1* amiRNA knockdown lines have low Cl^–^ accumulation in the shoot under low Cl^–^ supply

**Fig. 2.**
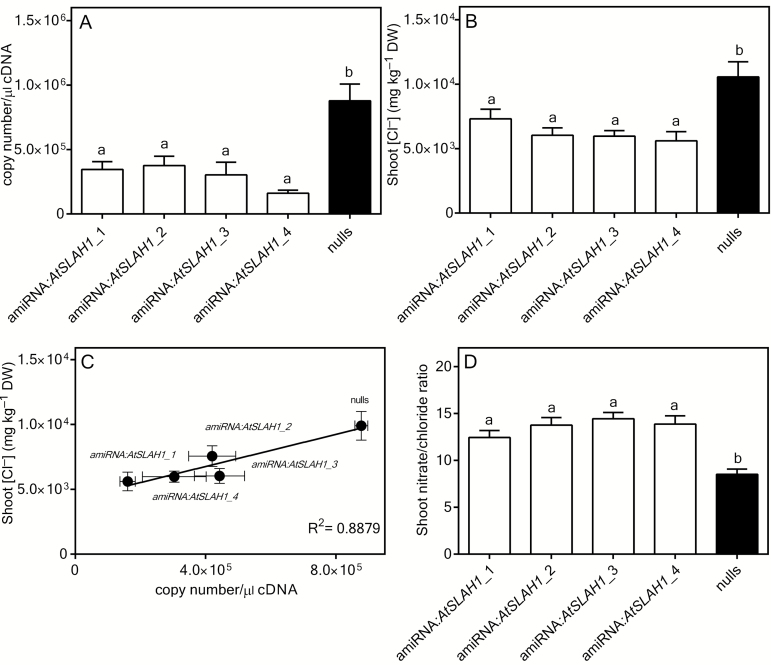
Under low Cl^–^ conditions, amiRNA:*AtSLAH1* mutants had significantly reduced expression levels of *AtSLAH1* and reduced shoot Cl^–^ compared with null segregants. Plants were grown hydroponically for 6 weeks in BNS containing 2mM NaCl (low Cl^–^ conditions). (A) *AtSLAH1* expression in roots of all amiRNA-*AtSLAH1* mutants (amiRNA:*AtSLAH1*_1, 2, 3 and 4) and null segregants (nulls). (B) Shoot Cl^–^ accumulation of amiRNA-*AtSLAH1* mutants and nulls under low Cl^–^ conditions. (C) Correlation between transcript level of *AtSLAH1* and shoot Cl^–^ concentration. (D) The shoot NO_3_^–^/Cl^–^ ratio in all amiRNA:*AtSLAH1* mutant and null segregant lines grown under low Cl^–^ conditions. Results are mean+SEM (*n*>8), except (C), which is ±SEM. Statistical differences determined by one-way ANOVA and Tukey’s test (*P*≤0.005); a and b represent statistically significant differences between data groups.

To investigate whether AtSLAH1 was involved in root-to-shoot anion transport, different Arabidopsis lines with an increase or decrease in *AtSLAH1* expression were generated. *Atslah1* knockout lines (FLAG_329G06) were ordered from the Versailles Arabidopsis Stock Centre. Homozygous lines were successfully identified (Supplementary Fig. S1); however, RT-PCR performed using *AtSLAH1*-specific primers (Supplementary Table S1) found that the expression of *AtSLAH1* was not abolished in these mutant lines (Supplementary Fig. S1C). Therefore, four independent amiRNA:*AtSLAH1* mutant lines were generated, which were named amiRNA:*AtSLAH1*_1 (two inserts), amiRNA:*AtSLAH1*_2 (two inserts), amiRNA:*AtSLAH1*_3 (three inserts) and amiRNA:*AtSLAH1*_4 (two inserts). Under low salt conditions (2mM NaCl), qRT-PCR showed that the transcript abundance of *AtSLAH1* in the root of all independent amiRNA lines was less than half of that found in the null segregants (*P*≤0.005) (Fig. 2A). In all amiRNA:*AtSLAH1* lines, the shoot Cl^–^ concentration was significantly lower than that of the null segregants under low salt conditions, being reduced by 30–47% (*P*≤0.005) (Fig. 2B). The expression level of *AtSLAH1* was plotted against shoot Cl^–^ concentration for each plant and a highly significant positive relationship was observed with an *R* ^2^ of 0.89. The shoot NO_3_^−^ concentration under low Cl^−^ supply was also determined and no difference was found between the mutants and the null segregants (Supplementary Fig. S3A), but the reduction in shoot Cl^–^ led to a significantly greater shoot NO_3_^–^/Cl^–^ ratio in all mutants when compared with the null segregants (Fig. 2D). No differences were found in shoot biomass in any of the amiRNA:*AtSLAH1* lines under low Cl^–^ conditions (Supplementary Fig. S3B). The shoot Na^+^ and K^+^ concentrations were determined in these plants and no differences were found between all mutants and null segregants (Supplementary Fig. S3C, D). The experiment was repeated using the same set of seeds, under the same treatments, and found to have similar results where shoot Cl^–^ accumulation was decreased in amiRNA:*SLAH1* lines (Supplementary Fig. S3E). Under high Cl^–^ supply, amiRNA lines had significantly reduced *AtSLAH1* transcript abundance compared with the null segregants in the same conditions (Supplementary Fig. S4A); however, the *AtSLAH1* expression in the null segregants was reduced by a quarter compared with low Cl^–^ conditions (Fig. 1A and Supplementary Fig. S4A). At the same time there was no difference in shoot Cl^–^ concentration between the knockdown plants and controls under high Cl^–^ supply (Supplementary Fig. S4B). This was presumably due to the native downregulation of *AtSLAH1* expression by high salt (Figs 1A and 2A and Supplementary Fig. S4A).

### Plants constitutively overexpressing *AtSLAH1* accumulate high Cl^–^ in the shoot under high Cl^–^ supply

**Fig. 3.**
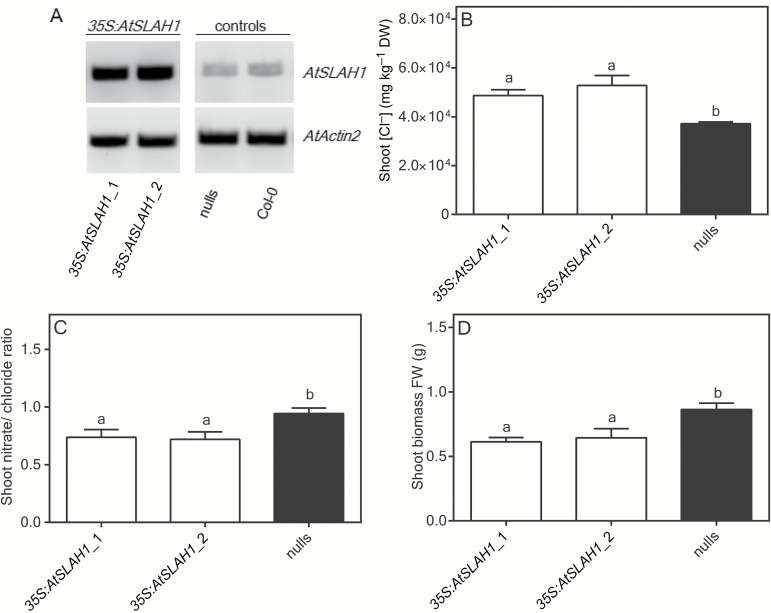
Under high Cl^–^ conditions, *35S:AtSLAH1* overexpression lines accumulated higher shoot Cl^–^ and showed reduced NO_3_^–^/Cl^–^ ratio compared with null segregants (nulls). Plants were grown hydroponically in BNS until 6 weeks old and then exposed to BNS containing 75mM NaCl (high Cl^–^ conditions) for 7 days. (A) Semi-quantitative RT-PCR of *35S:AtSLAH1* overexpression lines and nulls. (B) Shoot Cl^–^ concentration under high Cl^–^ conditions. (C) Shoot NO_3_^–^/Cl^–^ ratio. (D) Whole shoot biomass (fresh weight) as measured after high Cl^–^ treatment. Results are mean+SEM (*n*>6). Statistical differences determined by one-way ANOVA and Tukey’s test (*P*≤0.05); a and b represent statistically significant differences between data groups.

Plants with constitutive overexpression of *AtSLAH1* and their null segregants were selected by determining the presence or absence of the *AtSLAH1* transgene by PCR of genomic DNA. Relative expression of total *AtSLAH1* (consisting of both the native and the transgenic *AtSLAH1*) was then determined in root tissue by semi-quantitative RT-PCR. *AtSLAH1* was found to be highly expressed in both *35S:AtSLAH1* lines generated, whereas the null segregants had less abundant expression (Fig. 3A). When 75mM NaCl was applied to *35S:AtSLAH1*_1, *35S:AtSLAH1*_2 and null segregant lines for 7 days, significantly higher shoot Cl^–^ concentration accumulated in the overexpression lines when compared with the null segregants (*P*≤0.05), with no difference found between the two independent overexpression lines in shoot Cl^–^ concentration (Fig. 3B). The shoot NO_3_^–^ in both overexpression lines displayed no differences compared with null segregants (Supplementary Fig. S5A). Therefore, the increase in shoot Cl^–^ accumulation resulted in a decrease in shoot NO_3_^–^/Cl^–^ ratio in both overexpression lines under high Cl^–^ conditions (*P*≤0.05) (Fig. 3C). Both *35S:AtSLAH1* overexpression lines had significantly less whole shoot biomass when compared with the null segregants (*P*≤0.005) (Fig. 3D).

Under low Cl^–^ supply, the shoot Cl^–^ and NO_3_^–^ concentration of both *35S:AtSLAH1* overexpression lines was not significantly different from each other or the null segregants (Supplementary Fig. S5B, C), and the shoot biomass between all the genotypes was not significantly different (Supplementary Fig. S5D).

### Stelar-specific overexpression of *AtSLAH1* is correlated with increased shoot Cl^–^ accumulation under high Cl^–^ supply

**Fig. 4.**
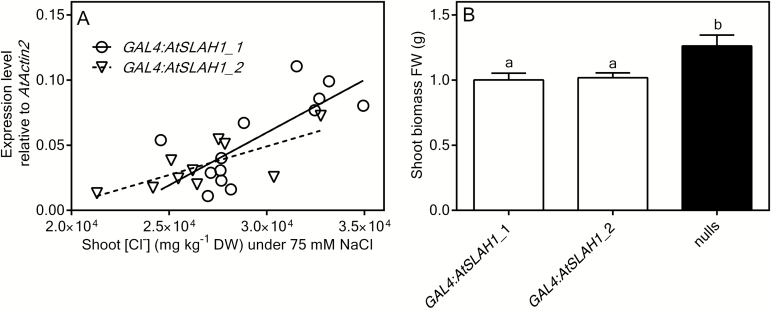
Stelar cell type-specific overexpression of *AtSLAH1* in E2586 significantly increased shoot Cl^–^ accumulation and reduced shoot biomass under high Cl^–^ conditions. (A) Correlation between shoot Cl^–^ accumulation and relative expression of *AtSLAH1* in *GAL4:AtSLAH1* overexpression lines under high Cl^–^ (75mM NaCl) supply. Open circles and solid line: *GAL4:AtSLAH1_*1; open triangle and dash line: *GAL4:AtSLAH1*_2. Plants were grown hydroponically in BNS for 6 weeks and then exposed to BNS containing 75mM NaCl (high Cl^–^ conditions) for 7 days. Correlation between shoot Cl^–^ concentration and the abundance of *AtSLAH1* in *GAL4:AtSLAH1_*1 (*R* ^2^=0.5805, *P* ≤ 0.005, significant deviation from zero), *GAL4:AtSLAH1_*2 (*R* ^2^=0.5395, *P* ≤ 0.005, significant deviation from zero). (B) Whole shoot biomass (fresh weight) as measured after high Cl^–^ treatment. Results are mean+SEM (*n*>6). Statistical differences determined by one-way ANOVA and Tukey’s test (*P*≤0.05); a and b represent statistically significant differences between data groups.

To further study the function of AtSLAH1 and avoid potential problems caused by non-targeted over expression in all cell types, root stelar cell-type specific over expression lines were generated following the method outlined by Møller *et al.* 2009. Two independent lines, named *GAL4:AtSLAH1_*1 and *GAL4:AtSLAH1_*2 were grown in hydroponics for 6 weeks before being supplied with 2 or 75mM NaCl for a further 7 days. Under high Cl^–^ conditions, both cell-specific overexpression lines had greater accumulation of Cl^–^ within the shoot; *AtSLAH1* expression and shoot Cl^–^ accumulation were again positively correlated (*R* ^2^=0.5, *P*≤0.01) (Fig. 4A). As with the constitutively overexpressing *AtSLAH1* plants, high salt treatment led to greater shoot Cl^–^ accumulation and no alteration in shoot concentration of Na^+^, K^+^ or NO_3_^–^ in the cell-specific overexpression lines compared to the null segregant lines (Supplementary Fig. S6A, B, C). Furthermore, the shoot biomass of the cell-specific overexpression lines was reduced under high salt treatment compared with the null segregants (Fig. 4B).

Under low Cl^–^ conditions, no significant differences in shoot Cl^–^ or NO_3_^–^ accumulation were identified between stelar-specific *AtSLAH1* overexpression lines and the null segregants (Supplementary Fig. S7A, B), but significantly less shoot Cl^–^ was accumulated in these plants in low Cl^–^ than when in high Cl^–^ treatment (Fig. 4A). Under low Cl^–^ conditions, the shoot biomass of both stelar-specific *AtSLAH1* overexpression lines was not significantly different from the null segregants (Supplementary Fig. S7C).

### *AtSLAH3* overexpression increases the shoot NO_3_^–^/Cl^–^ ratio under high and low Cl^–^

To compare the effects of *AtSLAH1* misexpression with that of a close homologue known to have a preference for NO_3_^–^ transport, we examined the phenotype of plants constitutively overexpressing *AtSLAH3*. In contrast to the greater Cl^–^ accumulation we observed in shoots of *AtSLAH1*-overexpressing plants, we observed a lower accumulation of shoot Cl^–^ under both high and low Cl^–^ supply in *AtSLAH3*-overexpressing plants (Supplementary Fig. S8A, B). The mean value for NO_3_^–^ concentration of the shoot was higher in the overexpression lines (Supplementary Fig. S8C, D), but not significantly compared with the null segregants under any condition tested, but coupled to the Cl^–^ data led to a significantly greater NO_3_^–^/Cl^–^ ratio in all conditions tested (Supplementary Fig. 8E, F).

### AtSLAH1 is likely to require additional co-factors to be active in *X. laevis* oocytes

*AtSLAH1* was expressed in *X. laevis* oocytes in an attempt to examine whether it could directly catalyse the transport Cl^–^ (Supplementary Materials and methods). No functional activity could be detected when *AtSLAH1* was expressed by itself (Supplementary Fig. S9). The AtSLAH1 homologue, AtSLAC1, was also found to be electrically silent in oocytes when expressed by itself, but when expressed with *sucrose non-fermenting-1-related protein kinase 2.6* (*SnRK2.6*) AtSLAC1 carried currents (Geiger *et al.*, 2009). To investigate whether a similar regulatory process was also required to trigger anion transport by AtSLAH1 in heterologous systems, *AtSnRk2.2* and *AtSnRk2.3* (root localized homologues of *AtSnRk2.6* that have their expression regulated by ABA; Yoshida *et al.*, 2006; Fujii and Zhu, 2009; Nakashima *et al.*, 2009) were co-injected with *AtSLAH1* in *X. laevis* oocytes (Supplementary Fig. S9F–H) but this resulted in no consistent activation of current, suggesting additional cofactors that regulate SLAH1 function still need to be identified.

## Discussion

### AtSLAH1 meets the predicted characteristics for a gene controlling Cl^–^ loading into the root xylem

Previous studies showed that *AtSLAH1* belongs to the *AtSLAC1* family; SLAC1 and SLAH3 underpin components of the slow type (S-type) anion conductance involved in anion efflux across the PM of stomatal guard cells in response to CO_2_ and O_3_ (Negi *et al.*, 2008; Vahisalu *et al.*, 2008; Geiger *et al.*, 2011; Demir *et al.*, 2013). The guard cell PM S-type anion conductance was found to be permeable to malate, Cl^–^ and NO_3_^–^ and its activation triggered by ABA (Schroeder and Hagiwara, 1989; Hedrich, 2012). AtSLAH3 and another family member, AtSLAH2, have been predicted to load NO_3_^–^ into the root stele (Maierhofer *et al.*, 2014; Zheng *et al.*, 2015). Whilst *AtSLAH1* is not usually expressed in guard cells it could complement the wild-type guard cell function of the *slac1* knockout when ectopically expressed, indicating it may encode or regulate a functional channel (Negi *et al.*, 2008; Vahisalu *et al.*, 2008); however, its true physiological functions are yet to be deciphered. Here, we confirmed that *AtSLAH1* was highly expressed in the Arabidopsis root and its expression was down-regulated strongly by ABA and NaCl treatment (Fig. 1). Previously AtSLAH1 was shown to be expressed in the root stele and pericycle of Arabidopsis roots and present on the PM (Brady *et al.*, 2007; Gifford *et al.*, 2008; Negi *et al.*, 2008; Supplementary Fig. S2). As the stelar-localized PM conductances capable of loading anions into the root xylem (and consequently the shoot) have been observed to be down-regulated by ABA (Gilliham and Tester, 2005) this suggests that AtSLAH1 could be involved in significant Cl^–^ loading of the root xylem. In contrast, we found that *AtSLAH3* transcript abundance was not reduced by salt or down-regulated by ABA (Fig. 1B). This coupled to the higher NO_3_^–^/Cl^–^ ratio of *AtSLAH3*-overexpressing plants (Supplementary Fig. S8E and F) suggests it does not, by itself, contribute to a significant proportion of Cl^–^ accumulation in the shoot.

### AtSLAH1 regulates Arabidopsis shoot Cl^–^ accumulation

As the ‘*Atslah1*’ T-DNA insertion mutant from the European Arabidopsis Stock Centre retained expression of *SLAH1*, we generated amiRNA lines that had reduced expression of *AtSLAH1* (Fig. 2A). Under low Cl^–^ supply (2mM NaCl), all *AtSLAH1* amiRNA lines had lower accumulation of Cl^–^ but not of NO_3_^–^ in the shoot (Fig. 2A, B and Supplementary Fig. S3A). There was also a strong positive correlation between *AtSLAH1* expression levels and shoot Cl^–^ (Fig. 2C), which suggests that *AtSLAH1* might play an important role in regulating Cl^–^ transport from root to shoot by affecting net loading of xylem vessels in the root. Whilst the shoot NO_3_^–^ concentration did not significantly alter when compared with the null segregants (Supplementary Fig. S3A), reduced *AtSLAH1* expression did lead to an increased shoot NO_3_^–^/Cl^–^ ratio due to a lower amount of Cl^–^ in the shoot (Fig. 2D).

Shoot Cl^–^ concentration was also examined in all amiRNA:*AtSLAH1* mutants exposed to high salt stress. No shoot Cl^–^ concentration differences were found between mutants and the null segregants under these conditions (Supplementary Fig. S4B). *AtSLAH1* expression is naturally decreased under high concentrations of NaCl (Fig. 1A). Therefore it is reasonable to suggest that the unchanged shoot Cl^–^ concentration in these plants was probably due to the endogenous down-regulation of *AtSLAH1* caused by high salinity. Therefore, the results of *AtSLAH1* overexpression lines might be expected to be more instructive for determining AtSLAH1 function under high salt conditions.

In *35S:AtSLAH1* overexpression lines we observed significantly increased shoot Cl^–^ accumulation compared with null segregants when grown under high Cl^–^ (75mM NaCl) (Fig. 3B); this is again consistent with AtSLAH1 being involved in xylem Cl^–^ loading. No difference in shoot NO_3_^–^, K^+^ or Na^+^ accumulation was observed between *35S:AtSLAH1* overexpression lines and null segregants under high Cl^–^ supply (Supplementary Fig. S5A, E, F) indicating that the role of AtSLAH1 is specific for Cl^–^. This translated into a reduced NO_3_^–^/Cl^–^ ratio in these lines compared with nulls (Fig. 3C). In overexpression lines, there was a concomitant decrease in shoot biomass compared with null segregant lines (Fig. 3C), suggesting that the level of Cl^–^ accumulated, and the reduction in NO_3_^–^/Cl^–^ ratio over this time period is suboptimal for growth. In many studies, the shoot K^+^/Na^+^ ratio is widely used to evaluate the plant’s salt tolerance: a higher K^+^/Na^+^ ratio value normally indicates a better salinity tolerance (Tester and Davenport, 2003). Due to the antagonism between Cl^–^ and NO_3_^–^ transport and the key roles of NO_3_^–^ in plant metabolism, it is reasonable to suggest that mechanisms that maintain high NO_3_^–^/Cl^–^ ratios might also be beneficial for improving salt tolerance.

These effects on shoot Cl^–^ accumulation, shoot NO_3_^–^/Cl^–^ ratio and shoot biomass were replicated in *AtSLAH1* root stelar cell-specific overexpression lines in high Cl^–^ conditions (in the cell types in which *AtSLAH1* is ordinarily expressed (Brady *et al.*, 2007; Gifford *et al.*, 2008; Negi *et al.*, 2008) (Fig. 4). This indicates that constitutive overexpression of *AtSLAH1* did not result in significant pleiotropic responses, which may be to do with the need for an unknown interacting partner in its native cell type for AtSLAH1 to be functional. What is important to note is that in *AtSLAH1* constitutively overexpressing plants and in the root stelar specific *AtSLAH1* overexpression lines in low Cl^–^ growth conditions, there was no growth phenotype compared with the null segregants (Figs 3 and 4). This linked to the fact that other ion contents (K^+^, Na^+^ or NO_3_^–^) were not altered in any conditions (Supplementary Figs S5 and S6), demonstrates that the growth of *AtSLAH1*-overexpressing plants was not altered by overexpression of the AtSLAH1 protein *per se*. Rather the inhibition of growth seen for *AtSLAH1*-overexpressing plants in high Cl^–^ was specifically due to the additional accumulation of Cl^–^ in the shoot (Figs 3 and 4).

Chloride accumulation in the shoot is a multigenic trait (White, 2001). We have shown that AtSLAH1 is likely to make up a component of this, as it has a significant effect on shoot Cl^–^ accumulation (~20–40% in various conditions), which suggests that other Arabidopsis anion transport proteins might be involved in root to shoot Cl^–^ transport. Recently, NFP2.4, a transport protein localized to the PM of stelar cells, was found to be important for Cl^–^ but not for NO_3_^–^ accumulation in shoots (Li *et al.*, 2016). AtCCC has also been shown to have an impact on shoot Cl^–^ accumulation (Colmenero-Flores *et al.*, 2007; Henderson *et al.*, 2015). However, AtCCC is predominantly localized to the Golgi and *trans*-Golgi network and so is unlikely to have a direct role in net loading of Cl^–^ into the xylem (Henderson *et al.*, 2015). Other candidates include transporters designated as NO_3_^–^ permeable, but which may also transport some Cl^–^, such as NRT1.5/NPF7.3, NRT1.8/NPF7.2 and SLAH3 (Lin *et al.*, 2008; Li *et al.*, 2010). A transcriptional comparison between the roots of good and poor Cl^–^-excluding grapevine rootstocks suggested further candidate genes for this multigenic trait including aluminium-acitivated malate transporters (ALMT), chloride channels (CLC) and their putative activating kinases (Henderson *et al.*, 2014). However, the true involvement of these candidate proteins requires that their substrates are resolved by functional assays. Therefore, the observed phenotypes in any single gene mutant are likely to be complicated by other functional proteins involved in Cl^–^ accumulation in the shoot.

### AtSLAH1 is likely to require unknown interacting proteins to function

No anion-mediated currents were identified when *AtSLAH1* cRNA was injected into *X. laevis* oocytes (Supplementary Fig. S9C, D). AtSLAC1, AtSLAH2 and AtSLAH3 are known to require protein kinases to be functional in oocytes (Geiger *et al.*, 2009; Vahisalu *et al.*, 2010; Brandt *et al.*, 2012; Demir *et al.*, 2013; Gutermuth *et al.*, 2013; Maierhofer *et al.*, 2014). For instance, when SLAC1 was expressed in *X. laevis* oocytes no clear anion currents were generated (Vahisalu *et al.*, 2008); however, co-expression with the protein kinase SnRk2.6 in oocytes was found to activate SLAC1 by phosphorylation of multiple serines in the SLAC1 hydrophilic N-terminal sequence (Geiger *et al.*, 2009; Lee *et al.*, 2009; Vahisalu *et al.*, 2010). This evidence suggests that a similar regulatory component is important for activating the S-type anion channels and may be required for SLAH1 activity. As SnRK2.6 has low expression in roots we concentrated on the root expressed members of the ABA-activated SNF1-related protein kinases 2, SnRK2.2 and SnRK2.3, which are both involved in ABA signalling pathways in roots (Yoshida *et al.*, 2006; Fujii and Zhu, 2009; Nakashima *et al.*, 2009). To identify whether a phosphorylation process initiated by a protein kinase was required to activate SLAH1 in oocytes, SnRK2.2/2.3 was co-injected with *AtSLAH1* into oocytes (Supplementary Fig. S9G–J). However, no activity was observed. Therefore, it is likely that AtSLAH1 requires additional factors for it to be active, or that it is itself a regulator of transport through its interaction with another transport protein.

## Conclusions

Manipulating *AtSLAH1* expression level in Arabidopsis resulted in significant alterations in shoot Cl^–^ concentrations suggesting AtSLAH1 is involved in Cl^–^ xylem loading in roots and the regulation of Cl^–^ accumulation in the shoot in response to salt stress. In contrast, overexpression of *AtSLAH3* resulted in relatively greater NO_3_^–^ content of the shoot under low and high Cl^–^ treatments. In the present work, heterologous expression studies were unable to distinguish whether AtSLAH1 acts directly as a transport protein or transport regulator. As *AtSLAH1* expression decreases under salinity and ABA, but *AtSLAH3* is still expressed, the relative capacity for roots to deliver NO_3_^–^ to the shoot is increased under saline conditions but the capacity for Cl^–^ loading is reduced. This will serve to maximize important NO_3_^–^ delivery to the shoot despite the increased competition from Cl^–^ during salinity stress. Therefore, it appears likely that AtSLAH1 and AtSLAH3 act in tandem to regulate NO_3_^–^ and Cl^–^ loading to the shoot and are both targets for manipulation in crops to improve salinity tolerance.

## Supplementary data

Supplementary data are available at *JXB* online.

Figure S1. *AtSLAH1* is still expressed in the *slah1* homozygous T-DNA insertion line FLAG_336C06.

Figure S2. The transcript level changes of *AtSLAH1* and *AtSLAH3* upon NaCl or ABA treatment.

Figure S3. Under low Cl^–^ conditions, the shoot NO_3_^–^, Na^+^, K^+^ concentrations and biomass were detected in all amiRNA:*AtSLAH1* mutants and null segregants (nulls).

Figure S4. Transcript abundance of *AtSLAH1* amiRNA containing lines (T_2_) and shoot Cl^–^ concentration under high Cl^–^ stress.

Figure S5. The shoot NO_3_^–^, Cl^–^ concentrations and shoot biomass were detected under low and high Cl^–^ supply in both *35S:AtSLAH1_*1 and *35S:AtSLAH1_*2, and null segregants (nulls).

Figure S6. Under high Cl^–^ conditions, the shoot Na^+^, K^+^ and NO_3_^–^ concentrations were detected in all *GAL4:AtSLAH1* overexpression lines and null segregants (nulls).

Figure S7. Under low Cl^–^ conditions, shoot Cl^–^, NO_3_^–^ concentrations and whole shoot biomass were detected in all *GAL4:AtSLAH1* overexpression lines and null segregant.

Figure S8. The shoot NO_3_^–^ and Cl^–^ concentrations were detected under low and high Cl^–^ supply in both *35S:AtSLAH3_*1 and *35S:AtSLAH3_*2, and null segregant (nulls) lines.

Figure S9. Electrophysiological characterization of *AtSLAH1* in *X. laevis* oocytes.

Table S1. Primers used for generating amiRNA:*AtSLAH1* constructs, for screening homozygous *Atslah1* T-DNA mutant lines and for cloning *AtSLAH1*/*AtSLAH3* from Arabidopsis.

Supplementary Data
