# Correction to: SLAH1, a homologue of the slow type anion channel SLAC1, modulates shoot Cl^−^ accumulation and salt tolerance in *Arabidopsis thaliana*

**DOI:** 10.1093/jxb/erac259

**Published:** 2022-07-18

This is a correction to: Jiaen Qiu, Sam W Henderson, Mark Tester, Stuart J Roy, Mathew Gilliham, SLAH1, a homologue of the slow type anion channel SLAC1, modulates shoot Cl^−^ accumulation and salt tolerance in *Arabidopsis thaliana*, *Journal of Experimental Botany*, Volume 67, Issue 15, August 2016, Pages 4495–4505, https://doi.org/10.1093/jxb/erw237

In the originally published version of this manuscript, an Australian Research Council funding code in the Acknowledgements section is incorrect. The relevant funding code should read “CE140100008” instead of “CE14010008”.

These details have been corrected only in this correction notice to preserve the published version of record.

