# Supplementary material for: SLAH1, a homologue of the slow type anion channel SLAC1, modulates shoot Cl− accumulation and salt tolerance in Arabidopsis thaliana
Source: J Exp Bot. 2016 Jun 23;67(15):4495–505. doi: 10.1093/jxb/erw237 (PMC4973733; doi:10.1093/jxb/erw237)
Supplement: Supplementary Data [file supp_67_15_4495__index.html]

SLAH1, a homologue of the slow type anion channel SLAC1, modulates shoot Cl− accumulation and salt tolerance in Arabidopsis thaliana — SLAH1, a homologue of the slow type anion channel SLAC1, modulates shoot Cl− accumulation and salt tolerance in Arabidopsis thaliana — Supplementary Data 

# SLAH1, a homologue of the slow type anion channel SLAC1, modulates shoot Cl− accumulation and salt tolerance in *Arabidopsis thaliana*

## Supplementary Data

Data files

- supplementary\_figures\_S1\_S9\_table\_S1.pdf - Supplementary Data
