# Supplementary material for: SLAH1, a homologue of the slow type anion channel SLAC1, modulates shoot Cl− accumulation and salt tolerance in Arabidopsis thaliana
Source: J Exp Bot. 2016 Jun 23;67(15):4495–505. doi: 10.1093/jxb/erw237 (PMC4973733; doi:10.1093/jxb/erw237)
Supplement: Supplementary Data [file supp_erw237_supplementary_figures_S1_S9_table_S1.pdf]

**Figure S1**

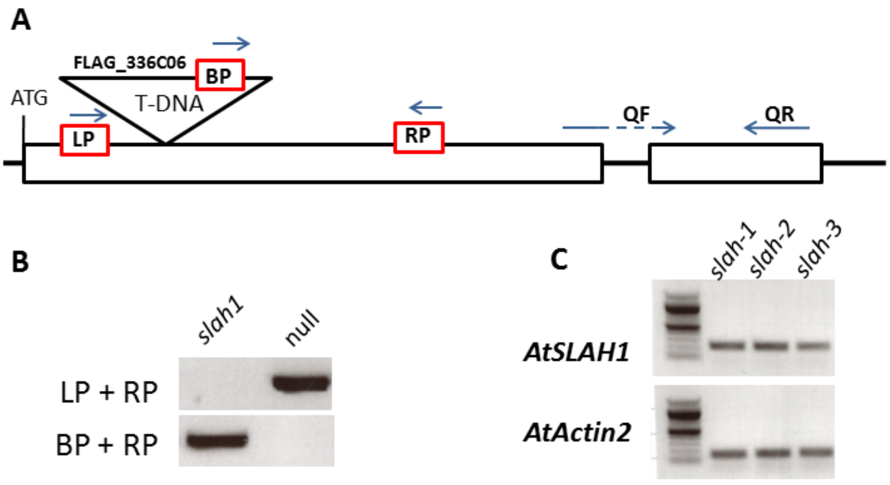


**Figure S1** *AtSLAH1* is still expressed in the slah1 homozygous T-DNA insertion line FLAG_336C06. (A) Map of the *slah1*T-DNA insertions in AtSLAH1 (At1g62280). LP, T-DNA left border primer; RP, T-DNA right border primer; BP, T-DNA border primer; QF, *AtSLAH1* q-RT-PCR forward primer; QR, q-RT-PCR reverse primer; (B) RT-PCR detection of T-DNA insertion in *slah1* mutant gDNA. Primers used were listed in Supplementary Table S1; (C) RT-PCR suggested that *AtSLAH1*'s expression is still detectable in mutant's cDNA, bands in upper panel were sequenced and showed that the amplified PCR products are *AtSLAH1* rather than non-specific bands. Primers used for detecting *AtSLAH1* expression were listed in Supplementary Table S1.

**Figure S2**

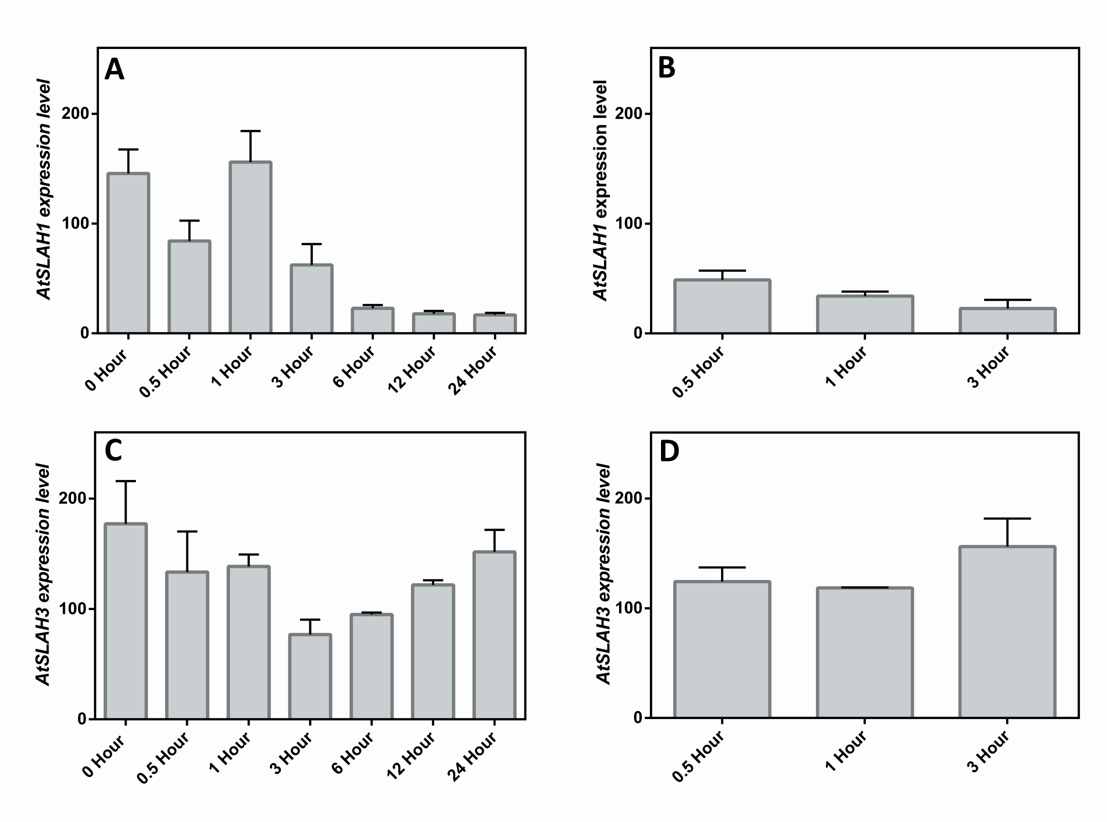


**Figure S2** The transcript level changes of *AtSLAH1* and *AtSLAH3* upon NaCl or ABA treatments. For NaCl treatment, 150 mM NaCl was applied for 0-24 hours and for ABA treatment, 20 μM ABA was applied for 0.5-3 hours. (A) and (B) *AtSLAH1*, (C) and (D) *AtSLAH3*. Data was extracted from Arabidopsis eFP Browser database. Results were presented as mean + SD (n = 3). Y axis: GCOS signal (adapted from Kilian *et al.*, 2007).

**Figure S3**

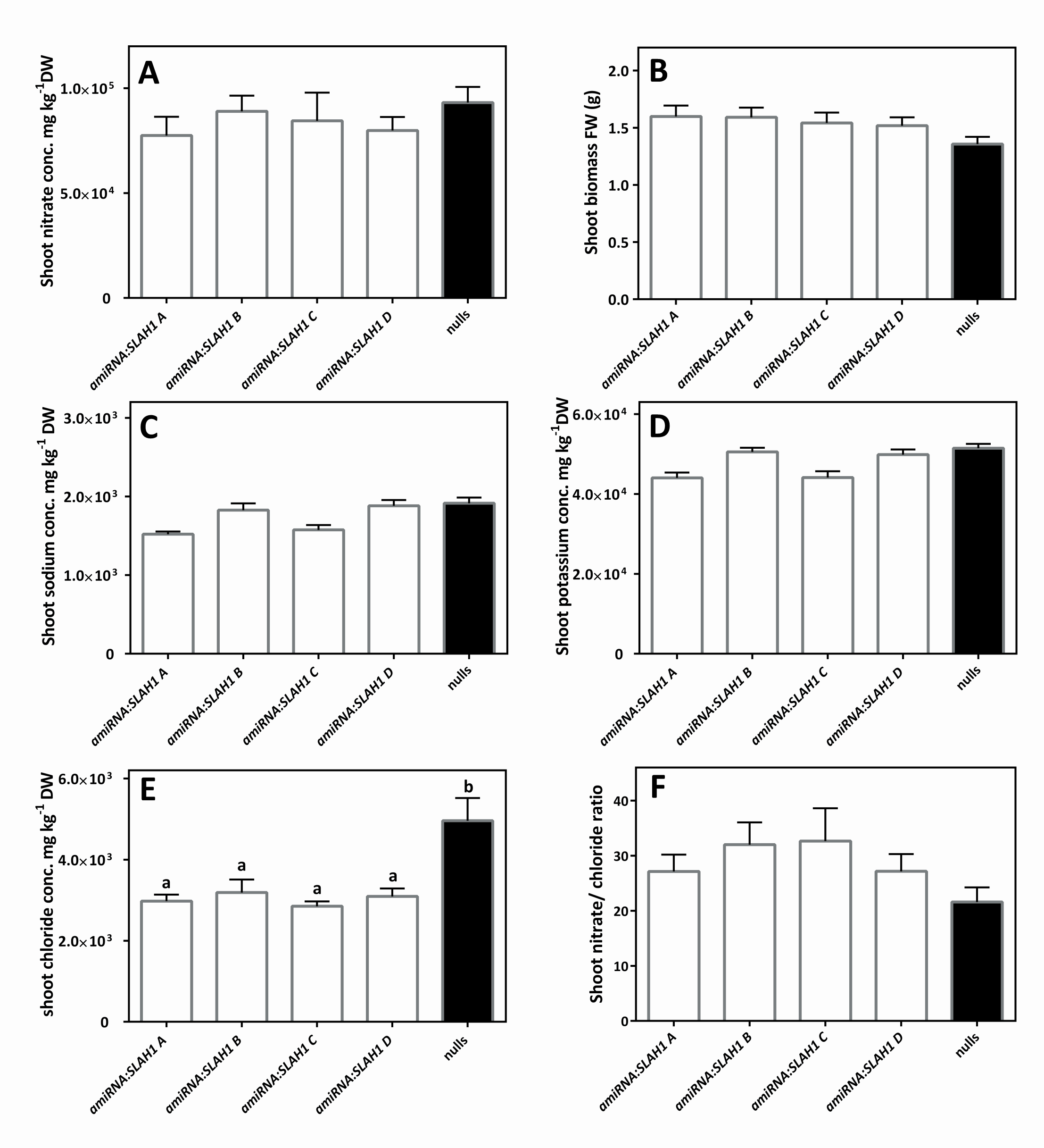


**Figure S3** Under low $Cl^-$ conditions, the shoot $NO_3^-$, $Na^+$, $K^+$ concentrations and biomass were detected in all *amiRNA:AtSLAH1* mutants and null segregants (nulls). Hydroponically grown plants (6 weeks old) supplied with BNS containing 2 mM NaCl (low $Cl^-$ conditions) were harvested at the same time point. (A) Shoot $NO_3^-$ accumulation of amiRNA:*AtSLAH1* mutants and nulls under low $Cl^-$ conditions. (B) Shoot biomass of amiRNA:*AtSLAH1* mutants and nulls under low $Cl^-$ conditions. (C) Shoot $Na^+$ accumulation of amiRNA:*AtSLAH1* mutants and nulls under low $Cl^-$ conditions. (D) Shoot $K^+$ accumulation of amiRNA:*AtSLAH1* mutants and nulls under low $Cl^-$ conditions. (E) Shoot $Cl^-$ accumulation of amiRNA:*AtSLAH1* mutants and nulls under low $Cl^-$ conditions in a replicate experiment. (F) Shoot $NO_3^-/Cl^-$ ratio of amiRNA:*AtSLAH1* mutants and nulls under low $Cl^-$ conditions in a replicate experiment. Results are presented as mean + SEM (n > 8). Statistically significant differences were determined by one-way ANOVA ($P \leq 0.005$). Letters a and b denote data groups that are statistically significantly different from each other.

**Figure S4**

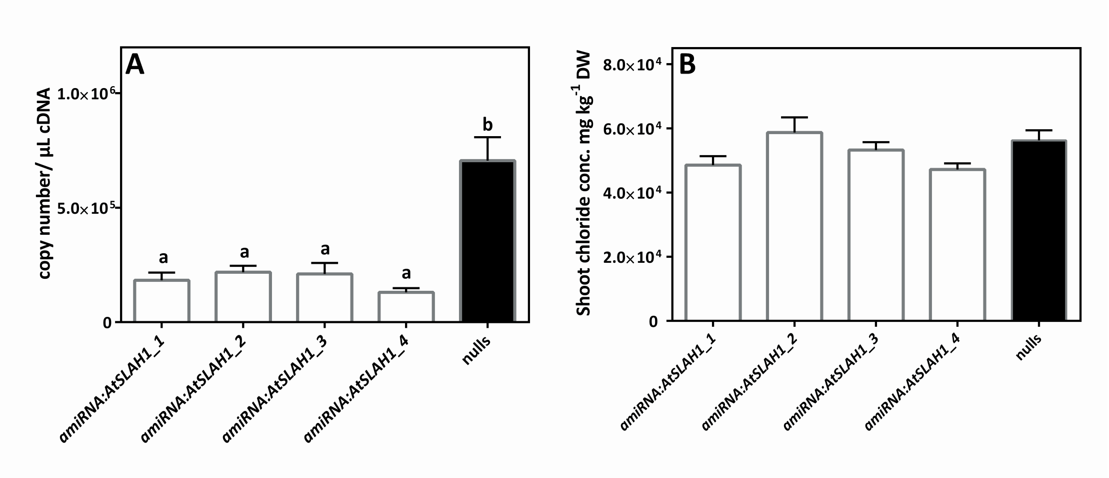


**Figure S4** Transcript abundance of *AtSLAH1* amiRNA containing lines ($T_2$) and shoot $Cl^-$ concentration under high $Cl^-$ stress. Hydroponically grown plants (5 weeks old) were treated with BNS containing 75 mM NaCl (high $Cl^-$ stress) for 7 days before harvest. (A) *AtSLAH1* transcript levels were determined in the root of all amiRNA-*AtSLAH1* mutants (amiRNA-*AtSLAH1*_1, 2, 3 and 4) and null segregants. (B) Shoot $Cl^-$ accumulation of amiRNA-*AtSLAH1* mutants and null segregants under high $Cl^-$ conditions. Results are presented as mean + SEM (n > 10). Statistically significant differences were determined by one-way ANOVA ($P \leq 0.005$). Letters a and b denote data groups that are statistically significantly different from each other.

**Figure S5**

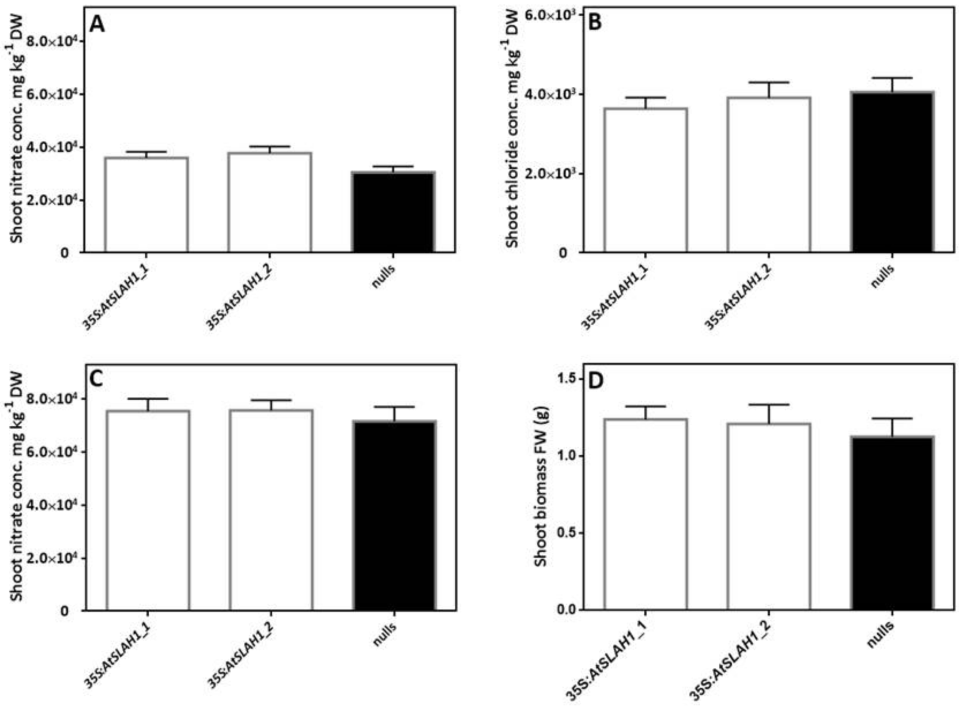


**Figure S5** The shoot $NO_3^-$, $Cl^-$ concentrations and shoot biomass were detected under high and low $Cl^-$ supply in both *35S:AtSLAH1-1* and *35S:AtSLAH1_2*, and null segregants (nulls). Hydroponically grown plants (6 weeks old) supplied with BNS containing 2 mM or 75 mM NaCl (low or high $Cl^-$ conditions) for 7 days were harvested at the same time point. (A) Shoot $NO_3^-$ accumulation of *35S:AtSLAH1* overexpression and null segregant lines under high $Cl^-$ conditions (75 mM NaCl). (B) Shoot $Cl^-$ accumulation of *35S:AtSLAH1* overexpression and null segregant lines under low $Cl^-$ conditions (2 mM NaCl). (C) Shoot $NO_3^-$ accumulation of *35S:AtSLAH1* overexpression and null segregant lines under low $Cl^-$ conditions (2 mM NaCl). (D) Whole shoot biomass of *35S:AtSLAH1* overexpression and null segregant lines under low $Cl^-$ conditions (2 mM NaCl). Results are presented as mean + SEM (n > 9). No significant differences were found using one-way ANOVA analysis or unpaired students t-tests.

**Figure S6**

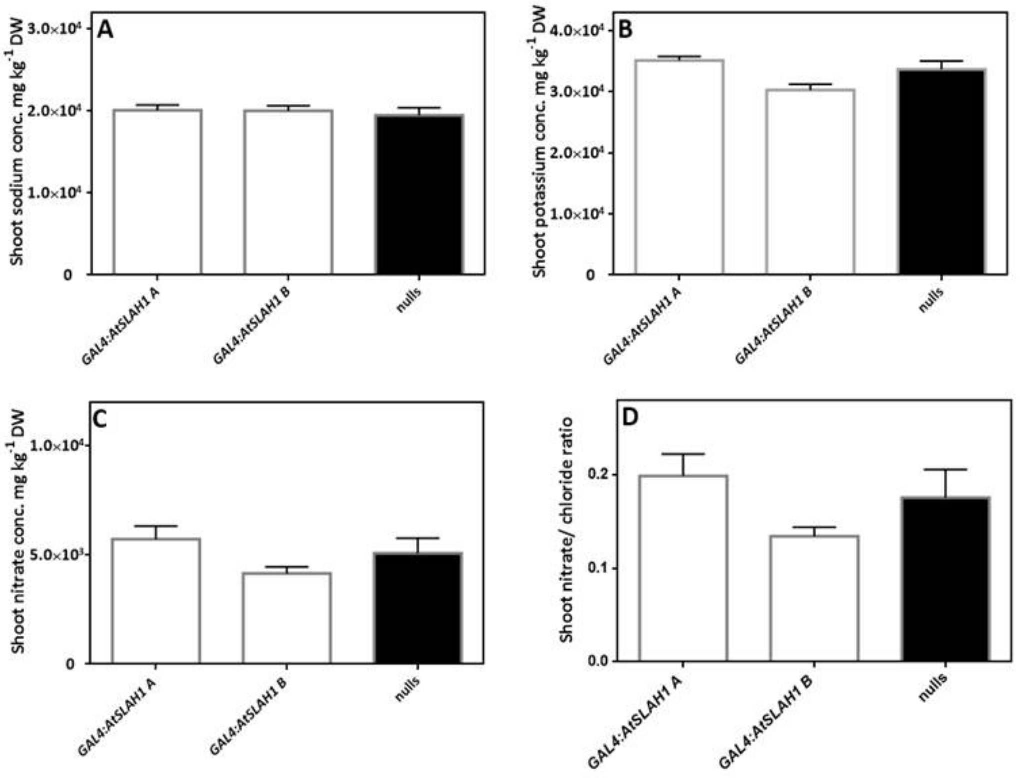


**Figure S6** Under high $Cl^-$ conditions, the shoot $Na^+$, $K^+$ and $NO_3^-$ concentrations were detected in all *GAL4:AtSLAH1* overexpression and null segregant (nulls) lines. Hydroponically grown plants (6 weeks old) supplied with BNS containing 75 mM NaCl (high $Cl^-$ conditions) were harvested at the same time point. (A) Shoot $Na^+$ accumulation of GAL4:*AtSLAH1* mutants and nulls under high $Cl^-$ conditions. (B) Shoot $K^+$ of GAL4:*AtSLAH1* overexpression and null lines under high $Cl^-$ conditions. (C) Shoot $NO_3^-$ accumulation of GAL4:*AtSLAH1* overexpression and null lines under high $Cl^-$ conditions. (D) Shoot $NO_3^-/Cl^-$ ratio of GAL4:*AtSLAH1* overexpression and null lines under high $Cl^-$ conditions. Results are presented as mean + SEM (n > 9). No significant differences were found using one-way ANOVA analysis or unpaired students t-tests.

**Figure S7**

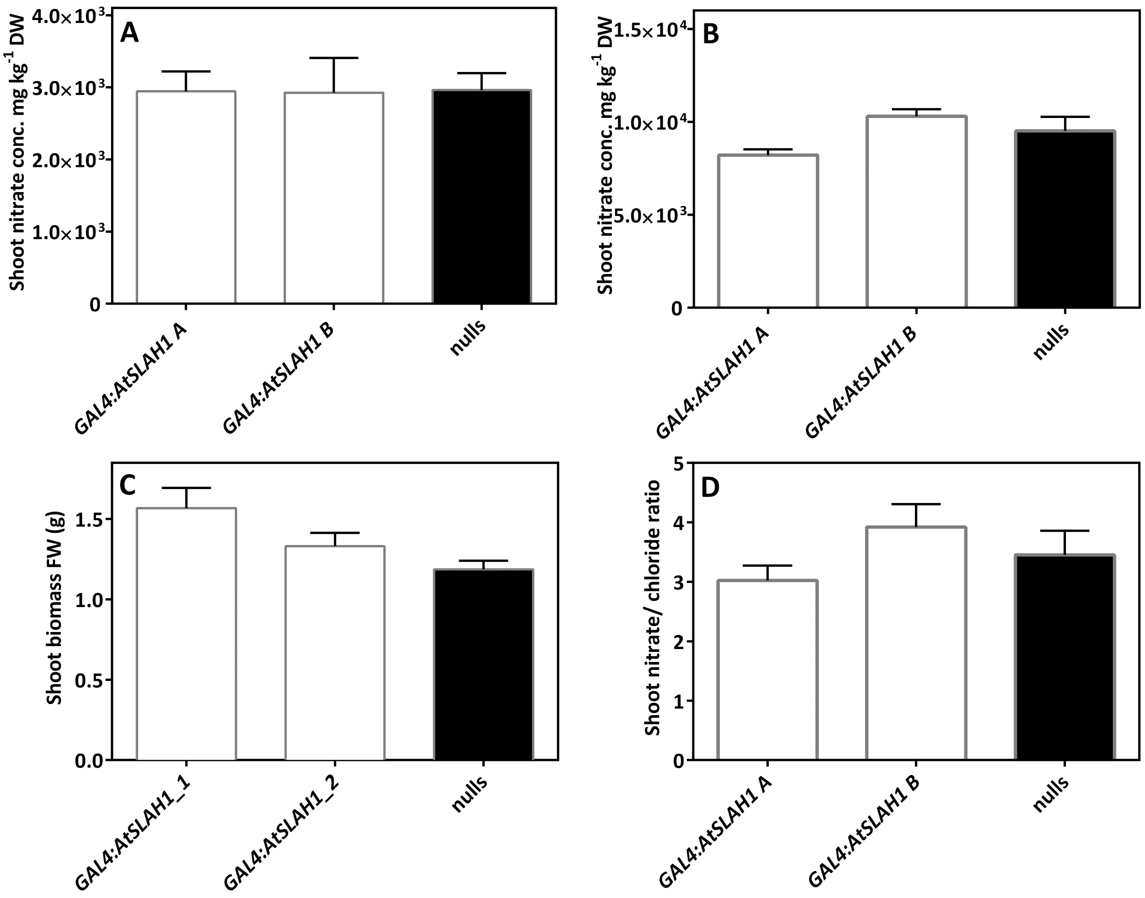


**Figure S7** Under low $Cl^-$ conditions, shoot $Cl^-$, $NO_3^-$ concentrations and whole shoot biomass were detected in all GAL4:*AtSLAH1* overexpression and null segregant (null) lines. Hydroponically grown plants (7 weeks old) supplied with BNS containing 2 mM NaCl (low $Cl^-$ conditions) were harvested at the same time point. (A) Shoot $Cl^-$ accumulation of GAL4:*AtSLAH1* overexpression and null lines under low $Cl^-$ conditions. (B) Shoot $NO_3^-$ accumulation of GAL4:*AtSLAH1* overexpression and null lines under low $Cl^-$ conditions. (C) Whole shoot biomass of GAL4:*AtSLAH1* overexpression and null lines under low $Cl^-$ conditions. (D) Shoot $NO_3^-/Cl^-$ ratio of GAL4:*AtSLAH1* overexpression and null lines under low $Cl^-$ conditions. Results are mean + SEM (n > 6). No significant differences were found using one-way ANOVA analysis or unpaired students t-tests.

**Figure S8**

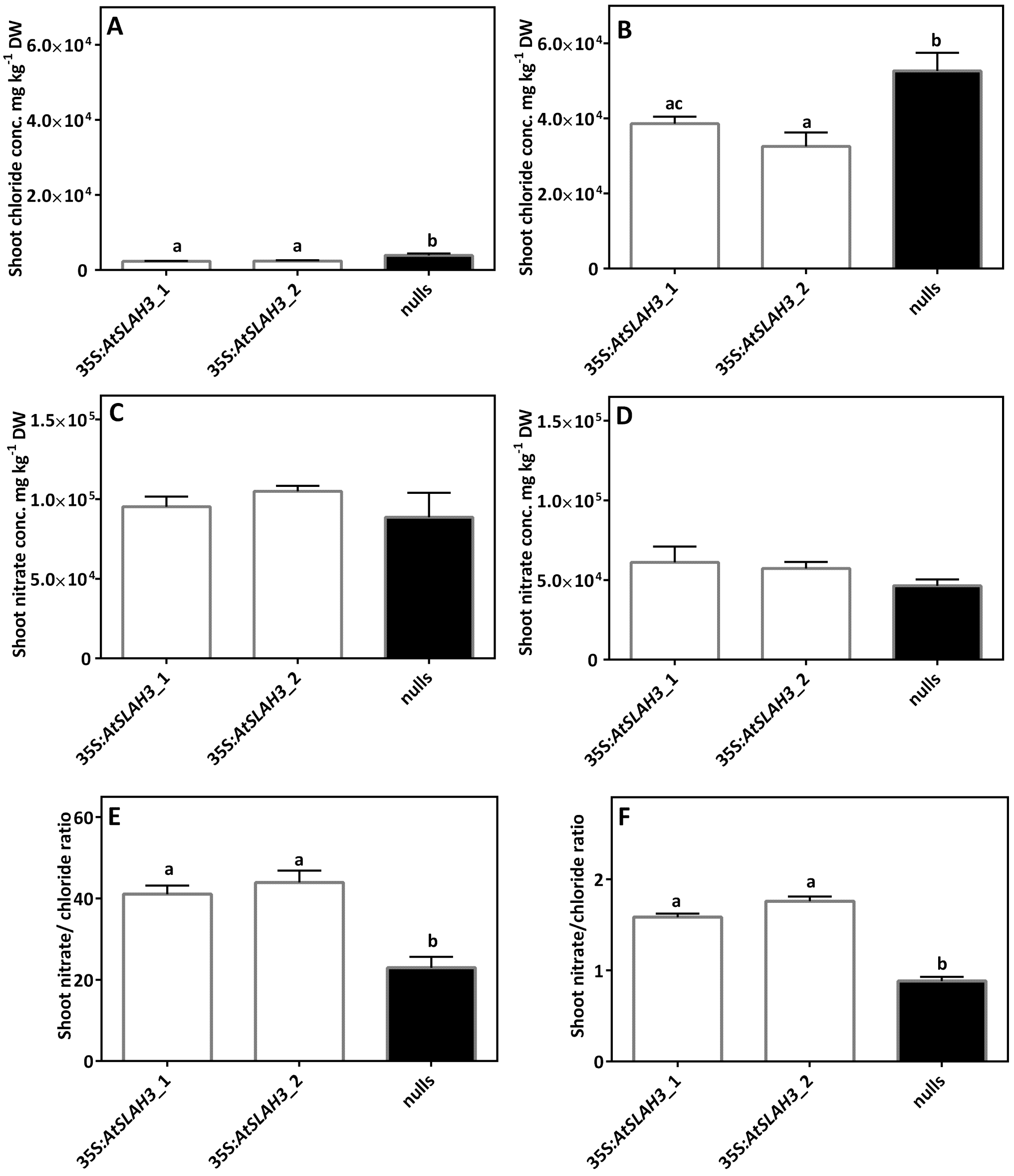


**Figure S8** The shoot $NO_3^-$ and $Cl^-$ concentrations were detected under low and high $Cl^-$ supply in both *35S:AtSLAH3-1* and *35S:AtSLAH3_2*, and null segregants (nulls). Hydroponically grown plants (6 weeks old) supplied with BNS containing 2 mM or 75 mM NaCl (low or high $Cl^-$ conditions) for 7 days were harvested at the same time point. (A) Shoot $Cl^-$ accumulation of 35S:*AtSLAH3* overexpression and null lines under low $Cl^-$ (2 mM) conditions. (B) Shoot $Cl^-$ accumulation of 35S:*AtSLAH3* overexpression and null lines under high $Cl^-$ (75 mM) conditions. (C) Shoot $NO_3^-$ accumulation of 35S:*AtSLAH3* overexpression and null lines under low $Cl^-$ conditions. (D) Shoot $NO_3^-$ accumulation of 35S:*AtSLAH3* overexpression and null lines under high $Cl^-$ conditions. (E) The shoot $NO_3^-/Cl^-$ ratio in all 35S:*AtSLAH3* overexpression and null lines grown under low $Cl^-$ conditions. (F) The shoot $NO_3^-/Cl^-$ ratio in all 35S:*AtSLAH3* overexpression and null lines grown under high $Cl^-$ conditions. Results are mean + SEM (n > 6). Statistical significance was determined by

one-way analysis of variance (ANOVA and Tukey test) ($P \leq 0.05$), a, b and c represent data groups that are statistically different from each other.

**Figure S9**

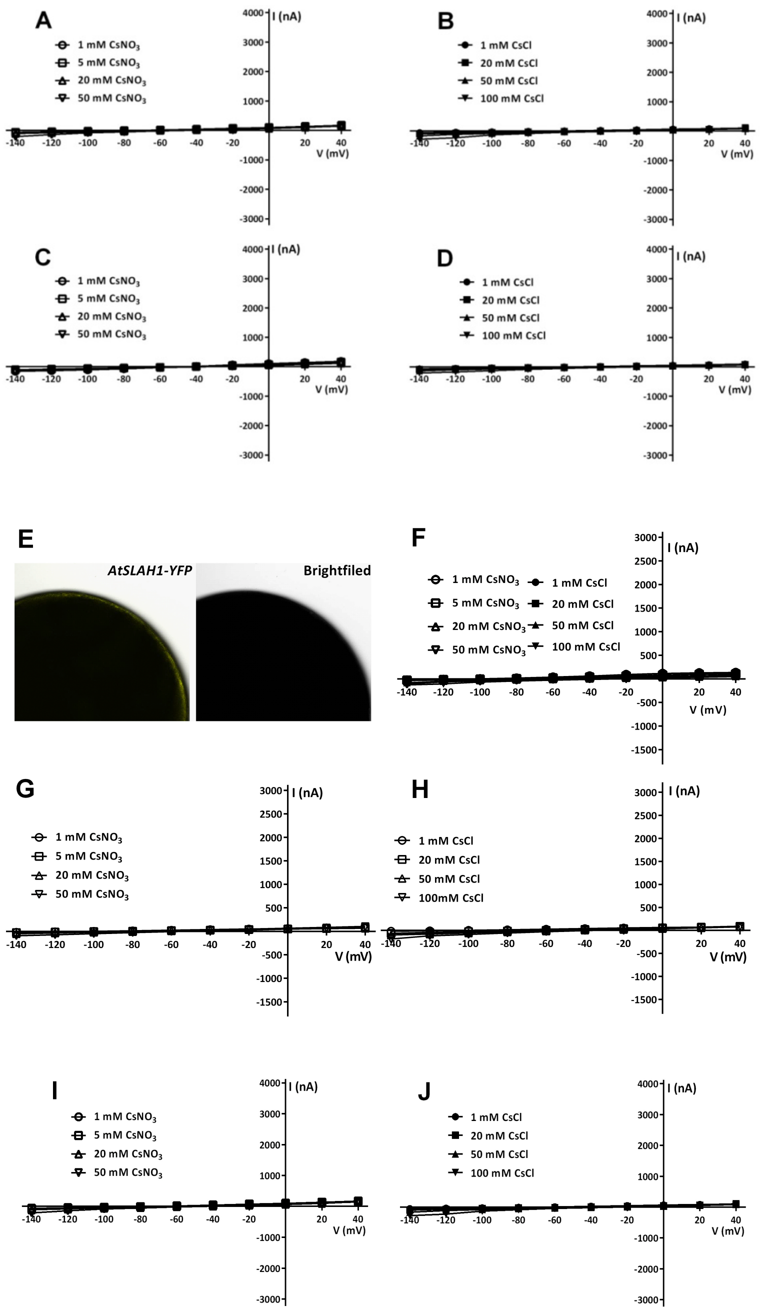


**Figure S9** Electrophysiological characterisation of *AtSLAH1* in *X. laevis* oocytes. Whole cell currents (steady state) in response to 3 second voltage pluses from +40 mV to -140 mV for *AtSLAH1* /*AtSLAH1+SnRK2.3* cRNA and nuclease-free water injected oocytes were recorded. (A) water injected oocytes perfused with 1, 5, 20 and 50 mM $CsNO_3$ at pH 7.5 (mean ± SEM, n = 4); (B) water injected oocytes perfused with 1, 20, 50 and 100 mM CsCl at pH 7.5 (mean ± SEM, n = 4); (C) *SLAH1* injected oocytes perfused with 1, 5, 20 and 50 mM $CsNO_3$ at pH 7.5 (mean ± SEM, n = 3); (D) *SLAH1* injected oocytes perfused with 1, 20, 50 and 100 mM CsCl at pH 7.5 (mean ± SEM, n = 4); (E) Transient expression of *AtSLAH1-YFP* in *X. Laevis* oocytes. (F) RNA free water injected oocytes perfused with 1, 5, 20 and 50 mM $CsNO_3$ or with 1, 20, 50 and 100 mM CsCl at pH 7.5 (mean ± SEM, n= 3); (G) *SLAH1-SnRK2.2* injected oocytes perfused with 1, 20, 50 and 100 mM $CsNO_3$ at pH 7.5 (mean ± SEM, n= 5); (H) *SLAH1-SnRK2.2* complex injected oocytes perfused with 1, 5, 20 and 50 mM CsCl at pH 7.5 (mean ± SEM, n= 5); (I) *SLAH1-SnRK2.3* injected oocytes perfused with 1, 20, 50 and 100 mM $CsNO_3$ at pH 7.5 (mean ± SEM, n= 5); (J) *SLAH1-SnRK2.3* complex injected oocytes perfused with 1, 5, 20 and 50 mM CsCl at pH 7.5 (mean ± SEM, n= 5); Data are presented without water subtraction.

**Supplementary Table S1**

Primers used for generating *amiRNA:AtSLAH1* constructs, for screening homozygous *Atslah1* T-DNA mutant lines and for cloning *AtSLAH1*/*AtSLAH3*/*AtSnRk2.2*/*AtSnRk2.3* from Arabidopsis.

| | |
|---|---|
| **Primers used for generating *amiRNA:AtSLAH1* mutant lines** | |
| Oligos for amiRNA "TAAAACGCTATTTGGTTCCGT" | |
| I miR-s | 5′ gaTAAAACGCTATTTGGTTCCGTtctctcttttgtattcc 3′ |
| II miR-a | 5′ gaACGGAACCAAATAGCGTTTTAtcaaagagaatcaatga 3′ |
| III miR*s | 5′ gaACAGAACCAAATACCGTTTTTtcacaggtcgtgatatg 3′ |
| IV miR*a | 5′ gaAAAAACGGTATTTGGTTCTGTtctacatatatattcct 3′ |
| Oligos for amiRNA "TTATGTCTAGTGTCGAGACTG" | |
| I miR-s | 5′ gaTTATGTCTAGTGTCGAGACTGtctctcttttgtattcc 3′ |
| II miR-a | 5′ gaCAGTCTCGACACTAGACATAAtcaaagagaatcaatga 3′ |
| III miR*s | 5′ gaCAATCTCGACACTTGACATATtcacaggtcgtgatatg 3′ |
| IV miR*a | 5′ gaATATGTCAAGTGTCGAGATTGtctacatatatattcct 3′ |
| **Primers used for screening *Atslah1* knockout lines (FLAG_329G06)** | |
| Forward (FP) | 5′ TGGCCTACAGACCTGAAAATG 3′ |
| Reverse (RP) | 5′ TTGGAATGACTTTGTGTGTGTG 3′ |
| Border (BP) | 5′ CTACAAATTGCCTTTTCTTATCGAC 3′ |
| **Primers used for detecting *AtSLAH1* expression in *Atslah1* knockout lines (FLAG_329G06)** | |
| Forward (QF) | 5′ TCTTCATGTCCCTGGTCTG 3′ |
| Reverse (RF) | 5′ ATTGCTGTTTGCTGCTGTC 3′ |
| **Primers used for cloning *AtSLAH1*, *AtSLAH3, AtSnRK2.2* and *AtSnRK2.3* from Arabidopsis** | |
| AtSLAH1 (F) | 5′ ATGGAAATTCCGAGGCAA 3′ |
| AtSLAH1 (R) | 5′ CTAGTTTTGGTTAGTCGCATTG 3′ |
| AtSLAH3 (F) | 5′ ATGGAGGAGAAACCAAACTAT 3′ |
| AtSLAH3 (R) | 5′ TTATGATGAATCACTCTCTTGAGT 3′ |
| AtSnRK2.2 (F) | 5′ ATGGATCCGGCGACTAAT3′ |
| AtSnRK2.2 (R) | 5′ TCAGAGAGCATAAACTATCTCTCC3′ |
| AtSnRK2.3 (F) | 5′ ATGGATCCGGCGACTAAT3′ |
| AtSnRK2.3 (R) | 5′ GAGAGCATAAACTATCTCTCCACT3′ |
